# Several miRNAs derived from serum extracellular vesicles are potential biomarkers for early diagnosis and progression of Parkinson’s disease

**DOI:** 10.1186/s40035-021-00249-y

**Published:** 2021-07-28

**Authors:** Shulei He, Lu Huang, Ci Shao, Tiejian Nie, Li Xia, Bozhou Cui, Fangfang Lu, Lin Zhu, Bolin Chen, Qian Yang

**Affiliations:** 1 Department of Experimental Surgery, Tangdu Hospital, The Fourth Military Medical University, Xi’an, 710038 China; 2 Department of Neurosurgery and Institute for Functional Brain Disorders, Tangdu Hospital, The Fourth Military Medical University, Xi’an, 710038 China; 3 School of Computer Science, Northwestern Polytechnical University, Xi’an, 710072 China

**Keywords:** Parkinson’s disease, Hoehn and Yahr stage, MicroRNAs, Extracellular vesicles, Biomarkers, Weighted gene co-expression network analysis

## Abstract

**Background:**

Blood-based test for predicting disease progression and early diagnosis of Parkinson’s disease (PD) is an unmet need in the clinic. The profiles of microRNAs (miRNAs) are regarded as potential diagnostic biomarkers for human diseases, whereas miRNAs in the periphery are susceptible to the influence of various components. MiRNAs enriched in serum extracellular vesicles (EVs) have demonstrated disease-specific advantages in diagnosis due to their high abundance, stability and resistance to degradation. This study was aimed to screen differentially expressed EV-derived miRNAs between healthy controls and PD patients to aid in diagnosis of PD.

**Methods:**

A total of 31 healthy controls and 72 patients with a diagnosis of PD at different Hoehn and Yahr stages in Tangdu Hospital were included. In total, 185 differentially expressed miRNAs were obtained through RNA sequencing of serum EVs as well as edgeR and *t*-test analyses. Subsequently, the weighted gene co-expression network analysis (WGCNA) was utilized to identify the commonly expressed miRNAs in all stages of PD by constructing connections between modules, and specifically expressed miRNAs in each stage of PD by functional enrichment analysis. After aligning these miRNAs with PD-related miRNAs in Human miRNA Disease Database, the screened miRNAs were further validated by receiver operating characteristic (ROC) curves and quantitative real-time polymerase chain reaction (qRT-PCR) using peripheral blood EVs from 40 more participants.

**Results:**

WGCNA showed that 4 miRNAs were commonly associated with all stages of PD and 13 miRNAs were specifically associated with different stages of PD. Of the 17 obtained miRNAs, 7 were validated by ROC curve analysis and 7 were verified in 40 more participants by qRT-PCR. Six miRNAs were verified by both methods, which included 2 miRNAs that were commonly expressed in all stages of PD and 4 miRNAs that were specifically expressed in different stages of PD.

**Conclusions:**

The 6 serum EV-derived miRNAs, hsa-miR-374a-5p, hsa-miR-374b-5p, hsa-miR-199a-3p, hsa-miR-28-5p, hsa-miR-22-5p and hsa-miR-151a-5p, may potentially be used as biomarkers for PD progression and for early diagnosis of PD in populations.

**Supplementary Information:**

The online version contains supplementary material available at 10.1186/s40035-021-00249-y.

## Background

Parkinson’s disease (PD) is characterized by loss of dopaminergic neurons in substantia nigra compact (SNc) and accumulation of Lewy bodies, and is diagnosed mainly based on clinical symptoms [1]. However, as non-motor alterations usually precede the onset of motor symptoms in PD, there is a great challenge to stratify PD patients, especially for early-stage PD patients among whom misdiagnosis is very common [2]. Progress has been made on early PD screening, with the use of neuroimaging methods such as DaT-SPECT imaging with (^123^I) ioflupane (DaTSCAN), which is a less expensive and more extensively available technique than positron emission tomography, and measurement of α-synuclein aggregation levels in cerebrospinal fluid (CSF) [3, 4]. Compared to the complication of DaTSCAN and invasive nature of CSF extraction, the blood-based test is rapid and safe, thus having potentials for use to detect biomarkers [5]. Apart from neurofilament light chain (NFL) which has been recognized as a marker for axon injury and may have been shown to be able to distinguish PD from atypical parkinsonian disorders (APDs) that show similar clinical symptoms with early PD [6], there are few validated biomarkers for screening PD in early stage and monitoring disease progression [7].

MicroRNAs (miRNAs) are a class of non-coding single-stranded small RNAs composed of 18–25 nucleotides and play major roles in cell differentiation, biological function and disease development [8]. Changes in the expression of many miRNAs can be used for differential diagnosis of PD from other neurodegenerative diseases (NDDs) such as frontotemporal dementia, amyotrophic lateral sclerosis and multiple system atrophy [9]. However, since miRNAs in the peripheral circulation are easily to be degraded by RNase, the capacity of circulating miRNAs to diagnose PD is restricted. MiRNAs can be selectively transported out of cells and attached to vectors such as Argonaute 2 protein as messengers for inter-cellular information transmission. The expression profile of miRNAs in the extracellular environment can reflect a pathological state [10], thus having higher diagnostic potential for NDDs.

Extracellular vesicles (EVs) are secreted by a wide range of cells and can be distinguished by size, mechanism of biogenesis and type of secretion (mainly three types) [11]. After derivation from the central nervous system (CNS), EVs in the peripheral blood carry a variety of proteins, lipids and nucleic acids (mRNAs and miRNAs), and play essential roles in biological information transmission and gene expression modification within recipient cells [12–14]. EVs can enrich and stabilize miRNAs to prevent them from degradation by nucleases that are widely present in body fluids, and pass freely through the blood-brain barrier, thus being able to directly reflect the conditions of CNS [15–17]. With advances in EV extraction and sequencing techniques, quantification of miRNA in serum-derived EVs by RNA sequencing (RNA-seq) and analysis of omics data by bioinformatics methods are increasingly used for the diagnosis of PD [16].

As PD is a multifactorial disease, it is critical to determine the process and key nodes of disease progression from the network level. Weighted gene co-expression network analysis (WGCNA), constructing the co-expression network to obtain key modules which consist of genes or miRNAs by clustering, is an unbiased approach to identifying core genes of diseases, intersections of biological pathways and key therapeutic targets of drugs [18]. It is more comprehensive and accurate than conventional expression analyses and is regarded as a typical algorithm for gene or miRNA screening.

In this study, we set out to screen for EV-derived miRNAs that are commonly expressed in PD and specifically expressed in each stage of PD, using RNA-seq and WGCNA approaches. The screened miRNAs were further verified by receiver operating characteristic (ROC) curves and quantitative real-time polymerase chain reaction (qRT-PCR), to demonstrate their potential as diagnostic biomarkers for PD.

## Materials and methods

### Recruitment of participants

Healthy controls and PD patients were recruited respectively from Physical Center and Department of Neurology of Tangdu Hospital (The Fourth Military Medical University, Xi’an, China). The exclusion criteria were: (1) recent fever, cognitive disorder, dyskinesia, severe head trauma, tumor, autoimmunity or active cold-like symptoms including cough, congestion, runny nose, headache, sore throat, etc.; (2) history of NDDs such as Alzheimer’s disease and Huntington’s disease; (3) severe primary disease involving the heart, brain, liver or kidney system; (4) alcohol abuse (> 14 units per day, a unit = 10 ml ethyl alcohol) or smoking addiction (> 5 cigarettes per day); and (5) atypical parkinsonism or haemolysis. A total of 103 participants were enrolled, including 31 healthy controls, 8 stage II, 42 stage III and 22 stage IV PD patients according to the Hoehn & Yahr (H&Y) scale [19]. The patients were diagnosed as idiopathic PD by H&Y stages without sex or age restrictions. All participants completed assessment with Mini-Mental State Examination (cognitive impairment score < 26) and Unified Parkinson’s Disease Rating Scale part III (on or off state). Informed consent was obtained from all participants. This study was approved by the Research Ethics Committee of Tangdu Hospital (K202011–05).

### Antibodies

The antibodies used for Western blotting were anti-CD63 (cat #A19023, Abclonal, Wuhan, China), anti-TSG101 (cat #ab125011, Abcam, Cambridge, UK), anti-CD81 (cat #ab109201, Abcam, Cambridge, UK), and anti-GAPDH (cat #D16H11, CST, Boston, USA).

### EV extraction and validation

Ten milliliters of fresh blood was collected from each participant. The blood sample was centrifuged at 3000 g for 10 min at 4 °C and the supernatant was transferred into another 15 ml centrifuge tube for another centrifugation at 10,000 g for 30 min with no break at 4 °C. After that, the supernatant was transferred to an ultracentrifuge tube and balanced with pure PBS for ultracentrifugation at 100,000 g for 70 min with no break at 4 °C. Part of the supernatant was discarded with 1.5 ml left to be resuspended and trimmed with pure PBS for next ultracentrifugation at 100,000 g for 70 min with no break at 4 °C. About 50 μl of liquid was left at the bottom and transferred to an RNase-free EP tube to be stored at − 80 °C.

After extraction from ultracentrifugation, the sample was added with 1× loading buffer of equal volume and heated at 95 °C for 10 min. Equal amounts of proteins from each sample quantified by BCA assay were separated in 10% SDS-PAGE and transferred onto PVDF membranes (cat # 03010040001, Roche, Basel, Switzerland). Then the membranes were blocked with 5% fat-extracted milk at room temperature for 2 h, and incubated with primary antibodies overnight at 4 °C. After that, the membranes were washed three times (5 min each) with TBST and incubated with secondary antibody for 2 h at room temperature. Protein bands were visualized using the electrochemiluminescence method.

Thirty microliters of EV suspension droplets extracted in the previous step were loaded onto a 200-mesh nickel formvar carbon-coated grid (Plano, Wetzlar, Germany) for 20 min. Then the grid was fixed with 1% glutaraldehyde in PBS and washed by distilled water. After that, the samples were negatively stained with 3% (*w*/*v*) phosphotungstic acid for 1 min and observed and photographed under a JEM-1230 transmission electron microscope (JEOL, Tokyo, Japan).

### RNA isolation and qRT-PCR assay

The extracted EVs were added with 1 ml of Trizol (cat #11667165001, Roche, Basel, Switzerland) and placed for 10 min to fully decompose. Then 200 μl of chloroform was added to the sample, vortexed for 20 s, placed for 3 min at room temperature, and centrifuged at 12,000 rpm for 15 min at 4 °C. The supernatant was extracted and transferred to another RNase-free EP tube on the ice, and a same volume of isopropyl alcohol was added and mixed upside down gently. After being placed for 30 min at − 20 °C, the samples were centrifuged at 12,000 rpm for 10 min at 4 °C. The supernatant was discarded, and the sediment was rinsed with 1 ml of 75% ethanol and centrifuged at 12,000 rpm for 10 min at 4 °C. Then the supernatant was discarded, and RNA was air-dried for 3 min in  super-clean bench and dissolved in 30 μl of DEPC H_2_O, and immediately used for reverse transcription to generate cDNA or stored at − 80 °C.

The isolated RNA was converted to cDNA using miScript II RT kit (cat # 218161, QIAGEN, Hilden, Germany) by reverse transcription according to the manufacturer’s protocol. The cDNA samples were diluted 5–10 times for qRT-PCR with Hieff® qPCR SYBR Green Master Mix (cat # 11201ES08, Yeasen, Shanghai, China). Primers are shown in Table S1. Raw Ct data were downloaded to calculate relative expression level of miRNAs using the 2^−ΔΔCt^ method, with U6 as a normalization control.

### RNA-seq and data preprocessing

Total RNA was used to construct small RNA libraries with the NEBNext® Multiplex Small RNA Library Prep Set and sequencing was performed by using the Illumina® HiSeq X Reagent Kit to obtain raw reads. After removing the adaptor sequences, the N-terminal nucleotide sequences, the low-quality (<Q20) sequences and sequences outside the range of 15–41 bp were filtered out with NGS QC Toolkit (version 2.3.3), fastx_toolkit (version 0.0.13) and cutadapt (version 1.14) respectively to obtain clean reads. Then, these reads were aligned with human reference genome (hg19, downloaded from UCSC website) using Rfam (version 10.0) database and Repbase database. Ultimately, Bowtie software was used to annotate the remaining sequences according to the mature miRNAs listed in the miRBase database, and the aligned sequences were identified as known miRNAs. The read counts under standardization in each final sample were used as the basis for miRNA expression statistics and subsequent differential expression analysis.

### Differential expression analysis and WGCNA

Differential expression analysis was performed using the edgeR package and the *t*-test method. A similar negative binomial generalized logarithm model from the edgeR package was applied. The model fitted the normalized read count value of each miRNA into the model, and a statistical test was performed for the given coefficient. miRNAs with *P* < 0.05 and |logFC| > 1 were identified as differentially expressed miRNAs. and for *t*-test, miRNAs with *P*-value< 0.05 and |T-statistic| > 2 were identified as differentially expressed miRNAs.

The correlation coefficient between miRNAs was used to construct the co-expression matrix, and further the power exponential adjacency function was used with its parameter β determined according to the principle of the scale-free network (β in stage II was 0.43; β in other stages were 0.05). The differences between nodes were measured and the layered cluster tree was generated by dynamic hybrid cutting. After stable miRNA modules were obtained, a threshold with low similarity was selected for module confirmation, which would be ultimately fused. All the modules were exported into a dot-edge file which was imported from Cytoscape, and color of the nodes was set to generate a visualized co-expression network diagram.

### Statistical analysis

Data (excluding qRT-PCR data) were nonnormally distributed as tested by the Shapiro-Wilk test. Data of participants’ demographic and clinical characteristics were analyzed with the nonparametric Kruskal-Wallis test. The area under the curve (AUC) of ROC curves was calculated to predict the diagnostic value of serum EV-derived miRNAs for early stage and progression of PD. The qRT-PCR data are presented as mean ± standard deviation (SD) from at least three independent experiments, and analyzed with one-way ANOVA with Dunnett’s multiple comparisons test. Statistical analyses were performed using GraphPad Prism 8 software. *P* < 0.05 was considered as statistically significant.

## Results

### EV-derived miRNAs and preprocessing of sequencing data

**Fig. 1:**
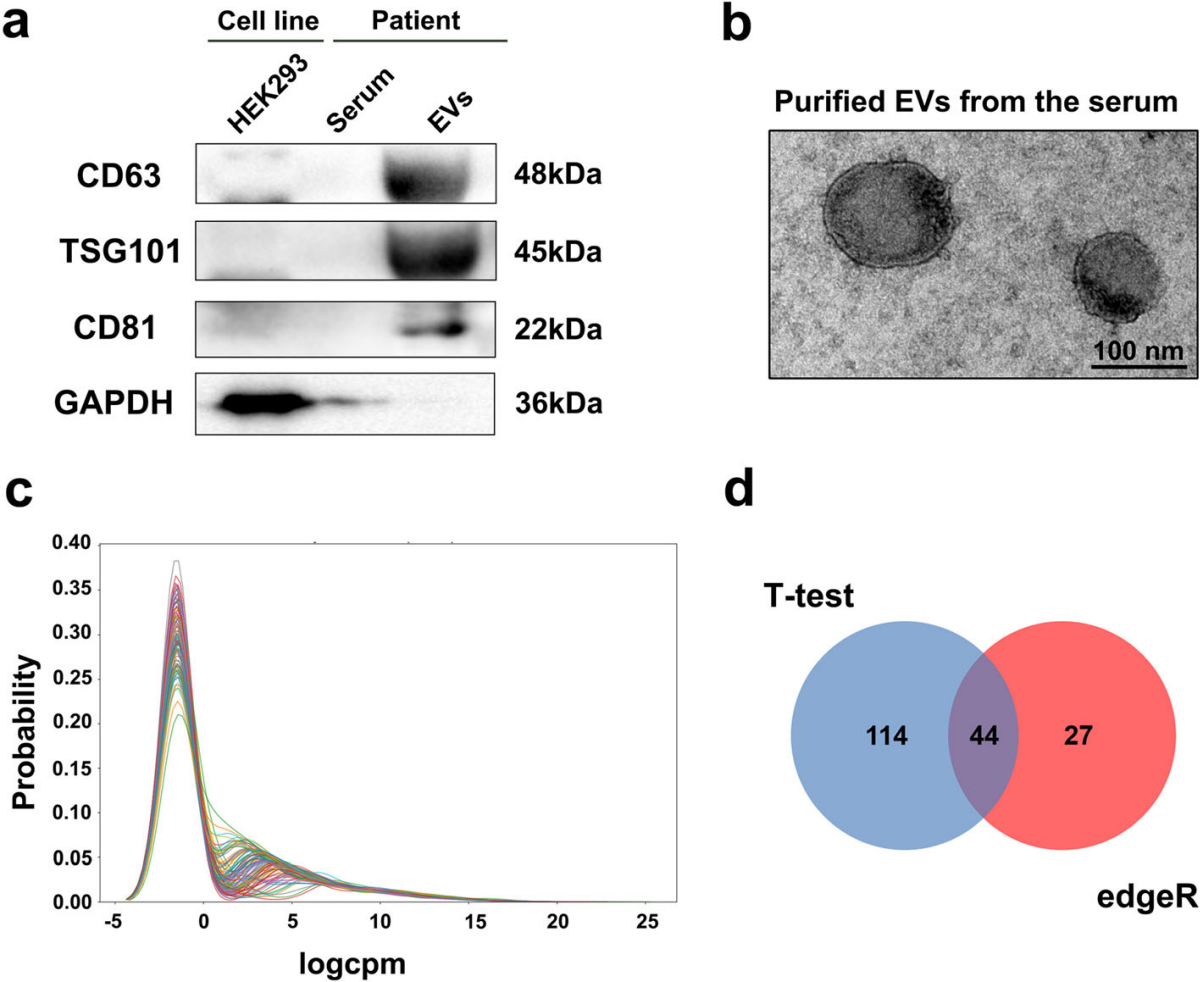
Quality control of EVs and differential expression analysis of miRNAs. **a** Verification of the extracted EVs by related biomarkers. EVs were extracted from serum samples of the participants according to the ultracentrifugation method, with HEK293 cells used as a control. Equal amounts of proteins were loaded for Western Blot with the indicated antibodies. **b** Verification of the extracted EVs by morphology and size. EVs were extracted according to ultracentrifugation method and the remaining 50 μl of liquid in the last step was observed by transmission electron microscopy. **c** Gaussian kernel density distribution diagram of 1486 miRNA expression in 103 samples. The ordinate represents the distribution density, the abscissas represents the logCPM value of miRNA expression in samples, and the curves with different colors represent different samples. **d** Venn diagram of differential expression analysis. edgeR and *t*-test methods were used to obtain the number of eligible miRNAs

The demographic and clinical characteristics of the participants are shown in Table 1. The presence of EVs was verified by western blot using antibodies for CD63, TSG101 and CD81, with cell-related marker (GAPDH) as a control for extracted vesicles (Fig. 1a). Transmission electron microscopy (TEM) demonstrated typical morphology and size of EVs (Fig. 1b).

**Table 1:** Demographic and clinical profiles of control and PD patients

Clinical parameters	Healthy controls	Stage II	Stage III	Stage IV	*P* value
Number of subjects	31	8	42	22	N/A
Age (years, mean ± SD)	63.94 ± 7.45	59.75 ± 7.55	61.62 ± 7.6	64.73 ± 8.14	0.2383
Age of onset (years, mean ± SD)	N/A	55 ± 7.53	53.6 ± 8.27	55.05 ± 10.89	0.6725
Sex (M/F)	17/14	5/3	26/16	12/10	0.0381
MMSE	N/A	26.63 ± 2.45	25.36 ± 2.32	24.18 ± 2.55	0.0392
UPDRS III	N/A	19.75 ± 2.90	25.1 ± 2.78	29.59 ± 4.45	< 0.0001

*M* male, *F* female, *MMSE* Mini-Mental State Examination, *UPDRS III* Unified Parkinson’s Disease Rating Scale part III, *N/A* not applicable

A total of 1486 miRNAs were acquired from the extracted EVs from 103 samples, and the expression density distribution was revealed (Fig. 1c). The trend of expression quantity was basically consistent among the samples, and the logCPM values of miRNA expression in most biological samples were distributed over − 5–15, which met the criteria for subsequent analysis. Then, differential expression analysis of these miRNAs was performed with edgeR and *t*-test for pairwise comparison between the control group, stage II, stage III and stage IV of PD. After considering the union of the two methods, miRNAs expressed in less than half of the samples were removed, and finally 185 miRNAs with differential expression were identified. Among them, 71 were obtained by edgeR and 158 by *t*-test, with 44 obtained by both methods (Fig. 1d).

### Identification of key modules containing miRNAs in all stages of PD

**Fig. 2:**
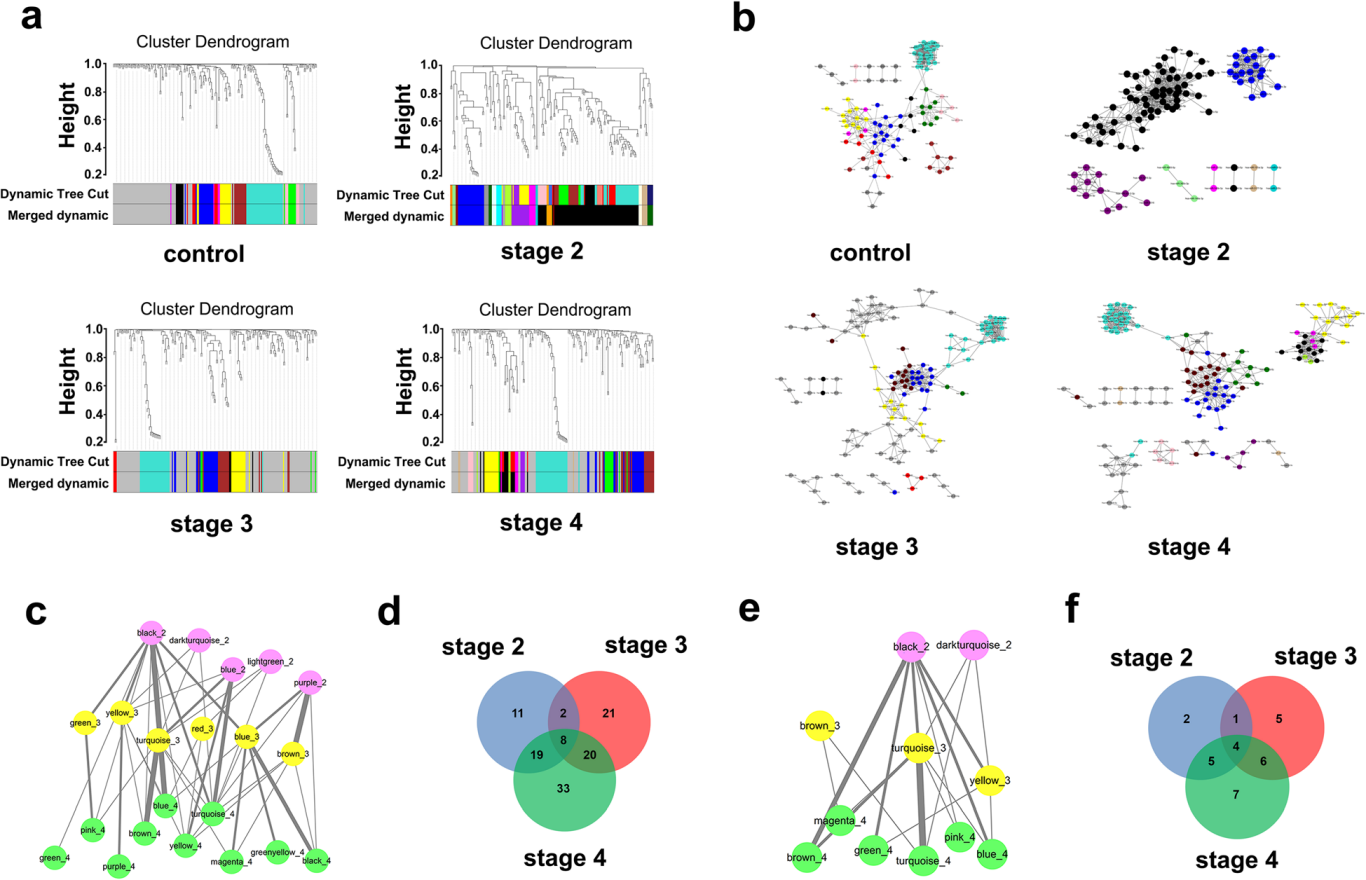
WGCNA of all the differentially expressed miRNAs. **a** Module clustering trees of control, stage II, stage III and stage IV constructed with all the differentially expressed miRNAs. Different colors represent different modules, and gray modules represent collections of miRNAs that were not assigned to any module. Dynamic tree cut: construction of the dynamic clustering tree; Merged dynamic: modules with high similarity were merged according to the minimum threshold of similarity. **b** The module network diagrams of control and stages II–IV obtained from the module clustering tree in **a**, by Cytoscape visualization. Different colors represent different modules. Different topological overlap thresholds were selected at different stages according to the size of the network and its accuracy, which were 0.05, 0.43, 0.05 and 0.05, respectively. Dark gray dots represent miRNAs that were not assigned to any module. **c** Module connection conditions of stage II (pink), stage III (yellow) and stage IV (green) after constructing the connections between modules of different stages. The strength of connection between different modules is expressed by the thickness of the line, with a higher thickness indicating a higher strength of connection. **d** Venn diagram of miRNAs in modules in **c**. **e** HMDD-aligned modules in **c**. **f** Venn diagram of miRNAs in modules in **e**

The miRNA co-expression networks were constructed with WGCNA among the 185 differentially expressed miRNAs. The clustering trees of modules for different stages of PD were visualized by Cytoscape (Fig. 2a). In total, 9 effective modules were obtained from the control group, 7 from stage II, 7 from stage III and 11 from stage IV of PD (Fig. 2b). A module connection was further established based on modules at different stages yet containing the same miRNAs, to identify key modules associated with the general pathogenesis of PD. The greater number of miRNAs shared by two modules, the stronger correlation between them. A network of 21 modules (Fig. 2c) comprising 114 miRNAs (Fig. 2d) was obtained. After discarding modules containing miRNAs that could not be aligned with those related to PD in Human miRNA Disease Database (HMDD), 11 key modules were identified, including 2 in stage II, 3 in stage III and 6 in stage IV (Fig. 2e), comprising 30 miRNAs (Fig. 2f). Among them, 4 miRNAs, hsa-miR-19b-3p, hsa-miR-374b-5p, hsa-miR-9-5p and hsa-miR-374a-5p, were commonly expressed in all stages of PD.

### Identification of key modules containing miRNAs in different stages of PD

**Fig. 3:**
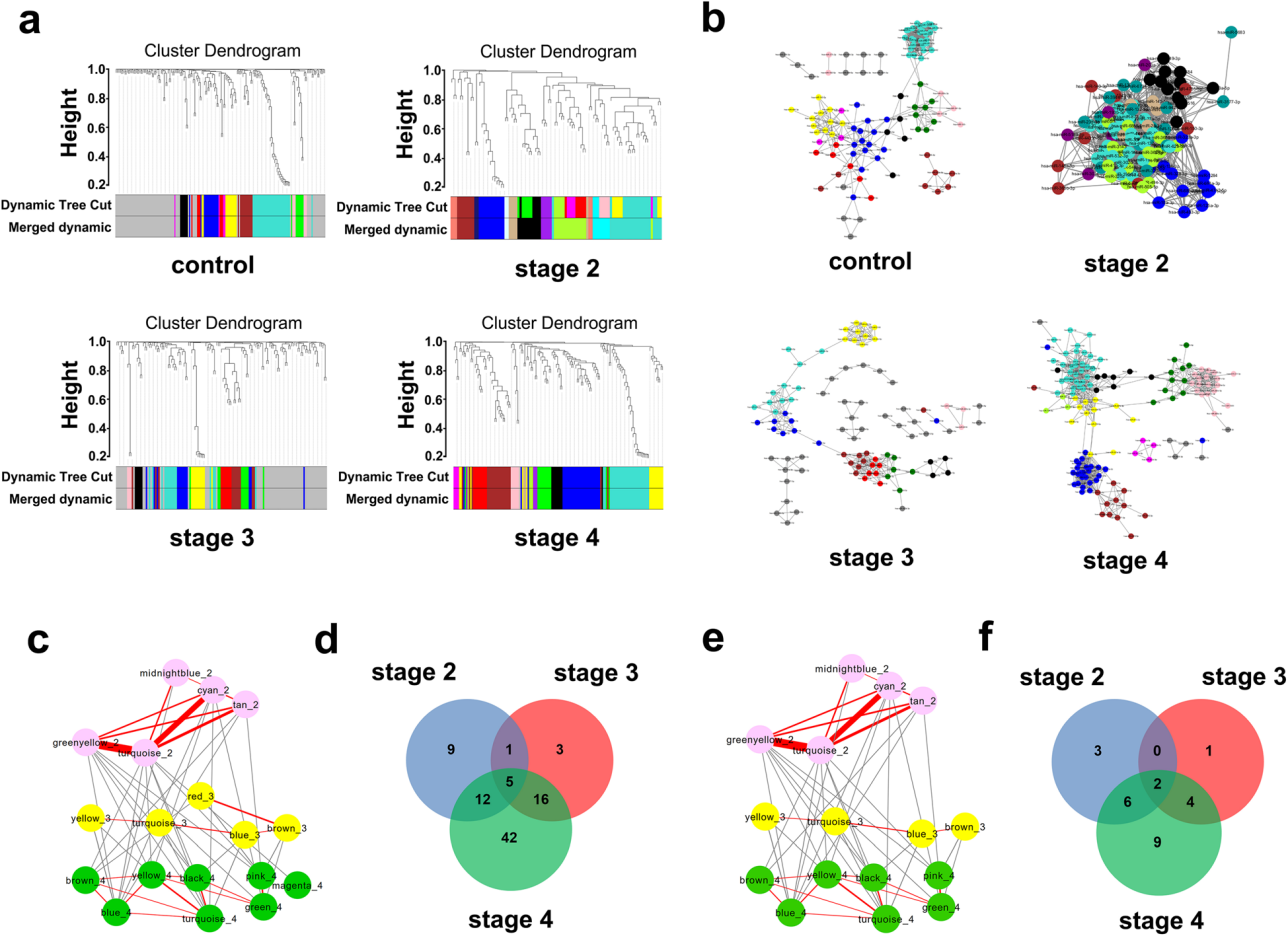
WGCNA of differentially expressed miRNAs in each stage. **a** Module clustering trees of control, stage II, stage III and stage IV constructed with differentially expressed miRNAs in each stage. Color indications as in Fig. 2. **b** The module network diagrams of control and stages II–IV obtained from the module clustering trees of **a**, by Cytoscape visualization. Different colors represent different modules. Different topological overlap thresholds were selected at different stages according to the size of the network and its accuracy, which were 0.05, 0.43, 0.05 and 0.05, respectively. Dark gray dots represent miRNAs that were not assigned to any module. **c** Enriched modules of stage II (pink), stage III (yellow) and stage IV (green) after functional enrichment analysis. The red line segment represents the module connections within the stage. The strength of connection between different modules is expressed by the thickness of the line, with a higher thickness indicating a higher strength of connection. **d** Venn diagram of contained miRNAs in modules of **c**. **e** HMDD-aligned modules of **c**. **f** Venn diagram of miRNAs contained in modules  in **e**

We also employed WGCNA to establish miRNA co-expression networks with the differentially expressed miRNAs between each two stages to clarify the unique characteristics of different stages of PD. The miRNA clustering tree at each stage was constructed to obtain stable miRNA modules (Fig. 3a). Finally, 9 effective modules were obtained from the control group, 11 from stage II, 8 from stage III and 9 from stage IV (Fig. 3b). We further conducted miRNA functional enrichment analysis using the TAM 2.0 database and selected the biological functions of these miRNAs in relation to the onset and progress of PD, at a false discovery rate < 0.05. In total, 18 modules (Fig. 3c) and 88 miRNAs (Fig. 3d) were obtained, which were involved in 15 biological functions with variations on functional enrichment among stages II–IV (Fig. S1). Subsequently, the detected miRNAs were aligned to the miRNAs associated with PD in HMDD. Only the modules containing miRNAs related to PD were selected as key modules. An inter-module network composed of 16 key modules was established, including 5 modules in stage II, 4 in stage III and 7 in stage IV (Fig. 3e), comprising 25 miRNAs (Fig. 3f). Among them, 13 miRNAs were expressed in a stage-specific manner, with 3 (hsa-miR-199a-3p, hsa-miR-195-5p and hsa-miR-28-3p) only appearing in stage II, 1 (hsa-miR-28-5p) only in stage III and 9 (hsa-miR-151a-3p, hsa-miR-183-3p, hsa-miR-29a-3p, hsa-miR-151a-5p, hsa-miR-205-5p, hsa-miR-29b-3p, hsa-miR-29c-3p, hsa-miR-30b-5p and hsa-miR-22-5p) only in stage IV.

### Evaluation of diagnostic value of the obtained miRNAs *via* ROC curves

**Fig. 4:**
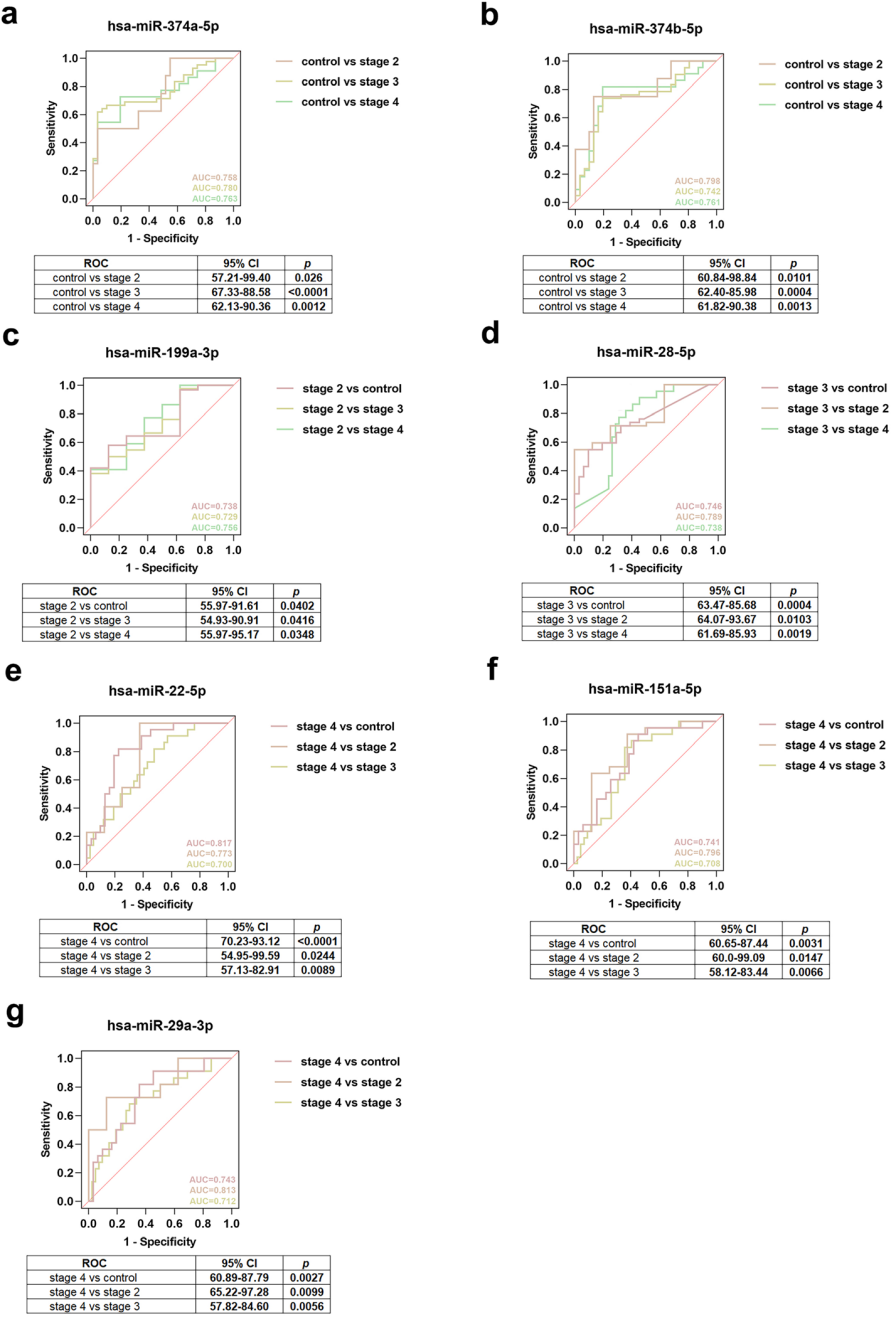
ROC curve analysis of screened miRNAs. **a** ROC curve of hsa-miR-374a-5p differentiating stage II, III or IV from control. **b** ROC curve of hsa-miR-374b-5p differentiating stage II, III or IV from control. **c** ROC curve of hsa-miR-199a-3p differentiating stage II from control, stage III or stage IV. **d** ROC curve of hsa-miR-28-5p differentiating stage III from control, stage II or stage IV. **e** ROC curve of hsa-miR-22-5p differentiating stage IV from control, stage II or stage III. **f** ROC curve of hsa-miR-151a-5p differentiating stage IV from control, stage II or stage III. **g** ROC curve of hsa-miR-29a-3p differentiating stage IV from control, stage II or stage III

Of the 4 miRNAs commonly expressed in all stages of PD and the 13 stage-specific miRNAs, 7 showed significant diagnostic value in all and different stages of PD. Among them, 2 miRNAs (hsa-miR-374a-5p and hsa-miR-374b-5p) distinguished stages II, III and IV from control. The AUCs of hsa-miR-374a-5p were 0.758 (95% confidence interval [CI]: 57.21–99.40, *P* = 0.026), 0.780 (95% CI: 67.33–88.58, *P* < 0.0001) and 0.763 (95% CI: 62.13–90.36, *P* = 0.0012) and the AUCs of hsa-miR-374b-5p were 0.798 (95% CI: 60.84–98.84, *P* = 0.0101), 0.742 (95% CI: 62.40–85.98, *P* = 0.0004) and 0.761 (95% CI: 61.82–90.38, *P* = 0.0013) (Fig. 4a, b). One miRNA (hsa-miR-199a-3p) distinguished stage II from control, stage III and stage IV with AUCs of 0.738 (95% CI: 55.97–91.61, *P* = 0.0402), 0.729 (95% CI: 54.93–90.91, *P* = 0.0416) and 0.756 (95% CI: 55.97–95.17, *P* = 0.0348) (Fig. 4c). One miRNA (hsa-miR-28-5p) distinguished stage III from control, stage II and stage IV with AUCs of 0.746 (95% CI: 63.47–85.68, *P* = 0.0004), 0.789 (95% CI: 64.07–93.67, *P* = 0.0103) and 0.738 (95% CI: 61.69–85.93, *P* = 0.0019) (Fig. 4d). Three miRNAs (hsa-miR-22-5p, hsa-miR-151a-5p and hsa-miR-29a-3p) distinguished stage IV from control, stage II and stage III. The AUCs of hsa-miR-22-5p were 0.817 (95% CI: 70.23–93.12, *P* < 0.0001), 0.773 (95% CI: 54.95–99.59, *P* = 0.0244) and 0.700 (95% CI: 57.13–82.91, *P* = 0.0089). The AUCs of hsa-miR-151a-5p were 0.741 (95% CI: 60.65–87.44, *P* = 0.0031), 0.796 (95% CI: 60.0–99.09, *P* = 0.0147) and 0.708 (95% CI: 58.12–83.44, *P* = 0.0066) and the AUCs of hsa-miR-29a-3p were 0.743 (95% CI: 60.89–87.79, *P* = 0.0027), 0.813 (95% CI: 65.22–97.28, *P* = 0.0099) and 0.712 (95% CI: 57.82–84.60, *P* = 0.0056) (Fig. 4e-g). For the remaining 10 miRNAs, 2 miRNAs were associated with stages II, III and IV from WGCNA but did not completely distinguish stage II, III and IV from control (Fig. S2a-b), 2 miRNAs were only associated with stage II from WGCNA but did not completely distinguish stage II from other stages (Fig. S2c-d), and 6 miRNAs were only associated with stage IV from WGCNA but did not completely distinguish stage IV from other stages (Fig. S2e-j).

### Validation of the obtained miRNAs *via* qRT-PCR

**Fig. 5:**
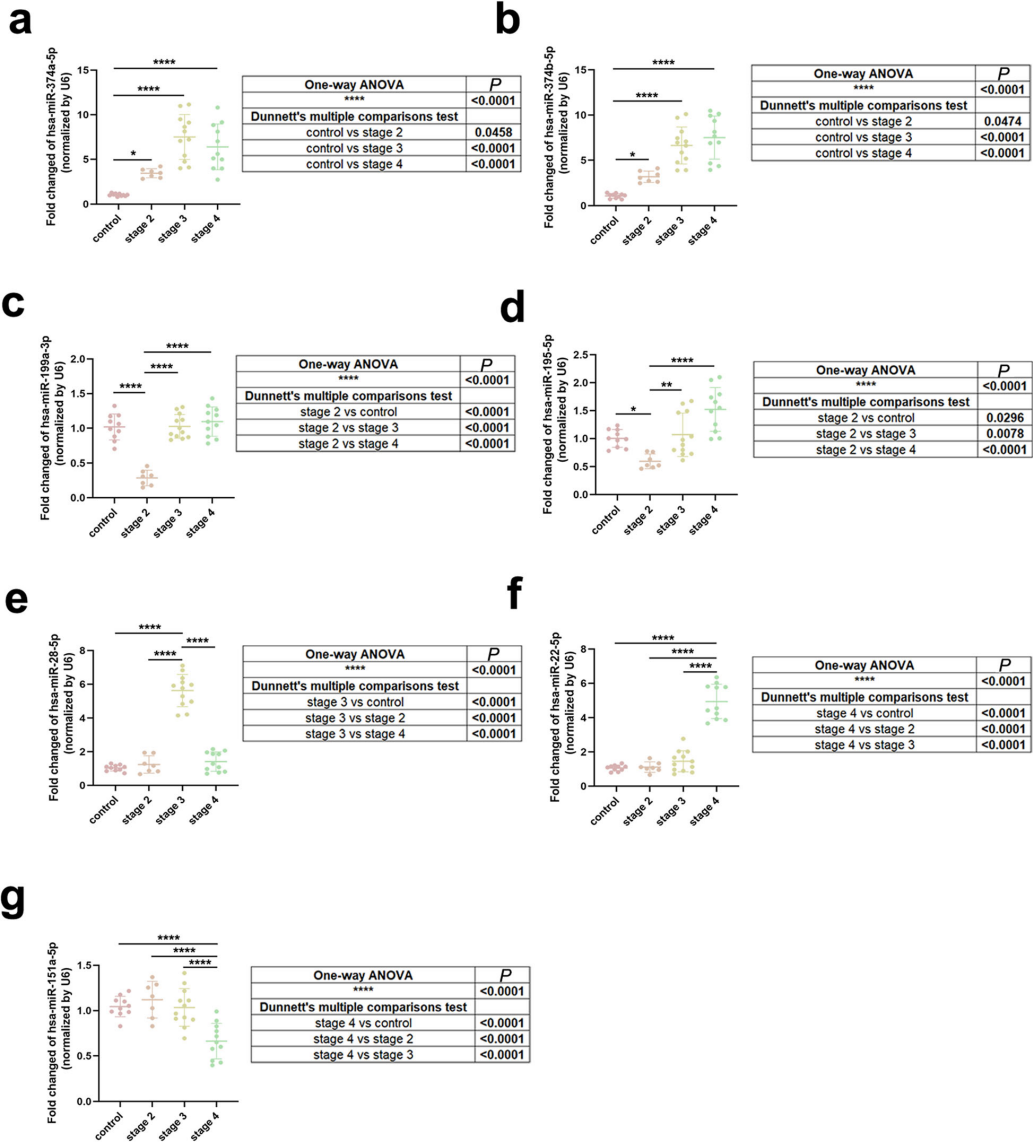
Verification of screened miRNA by qRT-PCR assay. **a–g** Expressions of hsa-miR-374a-5p (**a**), hsa-miR-374b-5p (**b**), hsa-miR-199a-3p (**c**), hsa-miR-195-5p (**d**), hsa-miR-28-5p (**e**), hsa-miR-22-5p (**f**), and hsa-miR-151a-5p (**g**) in stages II–IV. Data were analyzed by one-way ANOVA with Dunnett’s multiple comparisons test (mean ± SD; *n* = 40; *P < 0.05, **P < 0.01, ****P < 0.0001)

We collected serum samples and isolated EV-derived miRNAs for qRT-PCR assay from 40 more participants with same inclusion and exclusion criteria as previously described, including 10 healthy controls, 7 stage II, 12 stage III and 11 stage IV PD patients. The 17 miRNAs obtained from WGCNA and HMDD alignment were tested *via* qRT-PCR and 7 of them were verified to be commonly expressed in all stages or specifically expressed in different stages of PD. Among them, 2 miRNAs (hsa-miR-374a-5p and hsa-miR-374b-5p) were commonly up-regulated in stage II, III and IV (Fig. 5a-b), 2 miRNAs (hsa-miR-199a-3p and hsa-miR-195-5p) were specifically down-regulated in stage II (Fig. 5c-d), 1 miRNA (hsa-miR-28-5p) was specifically up-regulated in stage III (Fig. 5e), 1 miRNA (hsa-miR-22-5p) was specifically up-regulated, and 1 miRNA (hsa-miR-151a-5p) was specifically down-regulated in stage IV (Fig. 5f-g). For other 10 miRNAs, 2 miRNAs were associated with stages II, III and IV from WGCNA but did not show commonly differential expression in all stages of PD (Fig. S3a-b), 1 miRNA was only associated with stage II from WGCNA but did not show specific differential expression in stage II (Fig. S3c), and 7 miRNAs were only associated with stage IV from WGCNA but did not show specific differential expression in stage IV (Fig. S3d-j).

**Fig. 6:**
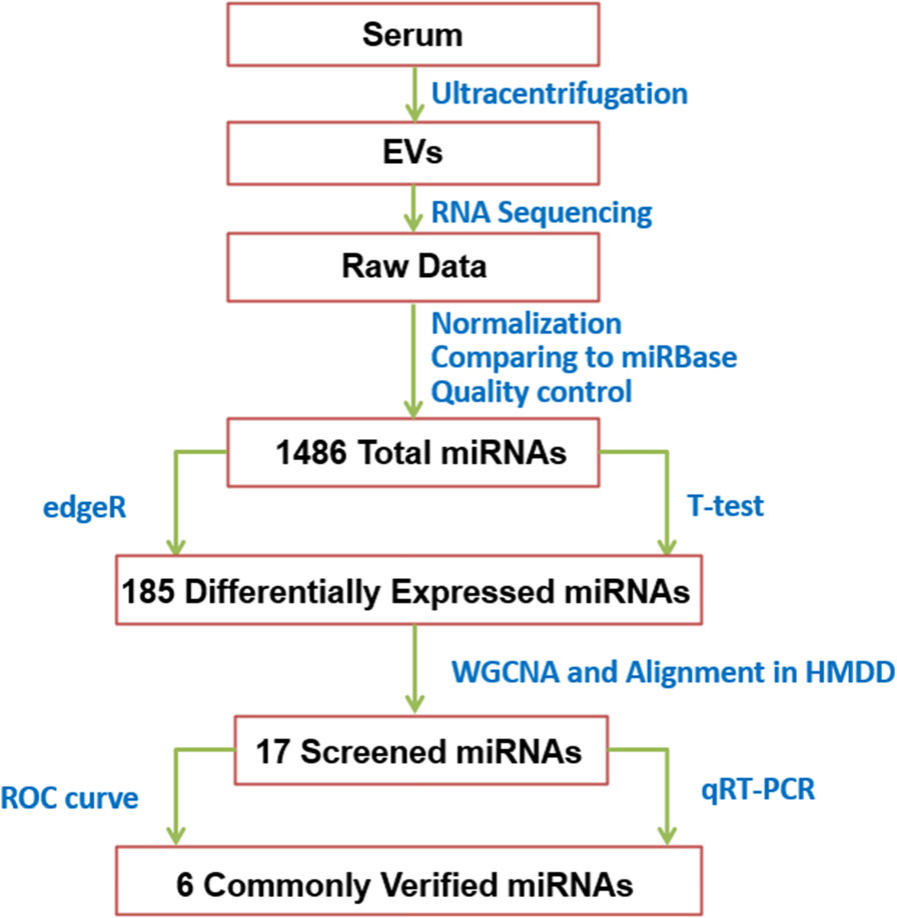
The flowchart of screening process for EV-derived miRNAs

The miRNAs validated by ROC curves and qRT-PCR assay are shown in Fig. S4. We also detected their expression using qRT-PCR in the sera of PD participants to check whether these 8 miRNAs were specifically expressed in EVs. Among them, only hsa-miR-195-5p and hsa-miR-29a-3p showed differential expression between some stages of PD, while other miRNAs showed no significant difference (Fig. S5a-h). The flowchart of EV-derived miRNAs’ screening process is shown in Fig. 6.

## Discussion

PD patients may not easily be screened out in the early stage although some non-motor symptoms including constipation, depression, REM sleep behavior disorder (RBD) and anosmia have been presented [19]. Classical diagnoses using biochemical methods are usually made with CSF, but the process of CSF collection may cause unpleasant feelings like anxiety. Therefore, non-invasive diagnostic testing with acceptable sensitivity and specificity is urgently needed for predictive early PD diagnosis. Plasma NFL has been reported to discriminate postural instability gait disorder (PIGD), one of the motor subtypes in early PD, from normal controls and motor global cognitive impairment in patients with PIGD [20]. Besides, various small RNA species obtained by the RNA-seq technology have demonstrated diagnostic and prognostic value for PD [21]. Over 7000 brain-derived long non-coding RNAs (lncRNAs), including their widespread transcript structure variations at the exons and splice junction levels as well as alteration of lncRNAs, have been identified from leukocytes of PD patients under pre- and post-DBS treatment and healthy controls, using a novel computational workflow *via* the RNA-seq technology and qRT-PCR validation [22]. The total level of circular RNAs (circRNAs) and their age-dependent accumulation in SNc are reduced based on the RNA-seq resource of several brain regions from PD participants. Particularly, circSLC8A1 and its target miR-128 have been found to be upregulated in the SNc of PD individuals, which may be related to the oxidative stress-related molecular function in PD [23]. A competing endogenous RNA (ceRNA) network with 92 dysregulated RNAs, containing 50 mRNAs, 25 miRNAs and 17 lncRNAs, constructed using the genome-wide RNA-seq data, is indicated to be a potential diagnostic and therapeutic biomarker for PD [24]. Besides, several novel miRNAs have also been discovered in the human post-mortem prefrontal cortex of PD samples utilizing the small RNA-seq technique, allowing for the identification of PD signature [25, 26]. MiRNAs are suitable candidates due to their insusceptibility to modification as well as the convenient and efficient detection method (qRT-PCR), but in the whole blood they are prone to interference from various components [27, 28], which calls for more applicable biomarkers for PD diagnosis.

Previous analysis has suggested that the disease-related constituents of EVs and EV-like vesicles isolated from blood or CSF samples of PD patients could be vital biomarkers for PD progression [29]. Unlike α-synuclein in serum that has low diagnostic accuracy for PD because of its high abundance in blood cells, the EV-derived α-synuclein secreted from the CNS into the serum is a potential biomarker for PD diagnosis with high specificity and sensitivity [30]. Moreover, the level of DJ-1 (PARK7) in serum EVs derived from the CNS is substantially higher than that in the plasma of PD patients [31]. Thus, the serum EVs could be an ideal tool for predictive PD diagnosis. With regard to the EV-derived miRNAs, EVs as RNase-protective vectors may contribute to the intercellular signal transmission of miRNAs and avoid influence from the peripheral environments on miRNAs [32–34]. The expression levels of miR-153 and miR-223 are significantly downregulated in the saliva of PD individuals, and their upstream heme oxygenase-1 in EVs could be transported from the CNS to the peripheral biofluids, all of them may be potential biomarkers for early PD [35, 36]. Upregulation of EV-derived let-7 in CSF together with activation of toll-like receptor 7 is also conducive to distinguish PD patients from controls [37, 38]. It is worth noting that any subtype of circulating EVs cannot be purified with currently available extraction methods, although direct TEM visualization of  typical concave and cup-shaped appearance of EVs has been widely used to analyze their size and morphology [12, 34, 39]. Other EM techniques such as cryo-electron microscopy which reveals a round shape of EVs are recently regarded as accurate detection measures for individual EVs in their native state [40, 41]. Therefore, new techniques for EV identification can improve the diagnostic value of EVs, and combined use of various specific proteins with small RNAs in EVs for early diagnosis of PD is an essential research direction [34].

As a method for mining gene or miRNA expression patterns in different samples, WGCNA can identify highly co-expressed modules to confirm biomarkers for a variety of diseases or to find key nodes in disease gene networks [18]. Moreover, WGCNA can not only adjust network construction and distinct module extraction by adopting suitable parameters with diagnostic values, but also identify the most closely correlated modules in disease progression [42–44]. From serum EVs of participants, we screened out 185 PD-related miRNAs by the edgeR and *t*-test methods, but some isolated miRNAs with false-positive expression have also been produced. Therefore, we identified key modules by WGCNA to screen for commonly expressed miRNAs in all stages and specifically expressed miRNAs in each stage of PD. Modules are a collection of several miRNAs with co-expression relationships, where there is a low rate of false positive miRNAs. We further conducted functional enrichment analysis to identify key modules that consisted of miRNAs present in different modules as a supplement to WGCNA, which makes the method more reliable in predicting vital nodes in progression of PD. However, considering the fact that the module integration in stage II was unsatisfactory, more cohorts of early-stage PD are needed in future efforts. Also, the uncertain origin of EV-derived miRNAs in our study may bring some limitations to the screen process by WGCNA. In the follow-up work, the origin of these miRNAs will be determined for more accurate use of WGCNA to prioritize miRNAs as potential biomarkers.

sHere, the miRNAs screened as diagnostic biomarkers were validated by ROC curves and qRT-PCR assay. Seven of the 17 miRNAs obtained from WGCNA and alignment in HMDD were identified by ROC curves to distinguish a specific stage of PD from others. Upon further validations *via* qRT-PCR with serum EVs from 40 more participants, we screened out 7 of 17 miRNAs that demonstrated stage-specific differential expression from WGCNA and alignment in HMDD. Furthermore, all the miRNAs identified by the two methods, totally 8 miRNAs, were detected with serum from the 40 participants. Our data showed that 6 miRNAs (hsa-miR-374a-5p, hsa-miR-374b-5p, hsa-miR-199a-3p, hsa-miR-28-5p, hsa-miR-22-5p and hsa-miR-151a-5p) were revealed by both methods and were considered as potential biomarkers for stage-specific diagnosis of PD. Regarding the miRNAs not aligned with PD-related miRNAs in HMDD, further studies and large-scale clinical trials are needed to verify their specificity and sensitivity. Meanwhile, although the existing approaches can isolate specific CNS-derived EVs in peripheral blood *via* related markers, such as NCAM and L1CAM [30], practical details and reproducibility of different protocols used in different laboratories are of crucial importance. Our study focused on the miRNA-screened pathway performed by WGCNA, ROC curve and qRT-PCR from 185 differentially expressed miRNAs, and found some miRNAs that could be potential biomarkers for early diagnosis and monitoring the progression of PD. The biological functions of these miRNAs in the development of PD need to be further explored in future studies.

## Conclusions

In summary, we collected serum EVs from healthy controls and PD patients, screened for miRNAs that are commonly expressed in all stages and specifically expressed in each stage of PD *via* WGCNA and HMDD alignment, and verified them by ROC curves and qRT-PCR assay. We found 6 serum EV-derived miRNAs (hsa-miR-374a-5p, hsa-miR-374b-5p, hsa-miR-199a-3p, hsa-miR-28-5p, hsa-miR-22-5p and hsa-miR-151a-5p) that could potentially be regarded as biomarkers for early diagnosis and progression of PD. The results lay foundation for clinical application of blood-based tests in PD diagnosis.

## Supplementary Information

**Additional file 1: Fig. S1.** The degree of miRNA functional enrichment in stages II–III. **Fig. S2.** ROC curve results of 10 miRNAs that did not show significant diagnostic value. **Fig. S3.** qRT-PCR results of 10 miRNAs whose specific expression failed to be verified by this analysis. **Fig. S4.** Venn diagram of miRNAs validated by ROC curve and qRT-PCR. **Fig. S5.** The expression of miRNAs validated by ROC curves and qRT-PCR assay, in the sera of PD participants. **Table S1.** Primer sequences for qRT-PCR.

## Data Availability

All data generated or analyzed during this study are included in this article.

## Abbreviations

PD: Parkinson’s disease
miRNA: MicroRNA
EVs: Extracellular vesicles
H&Y: Hoehn & Yahr
RNA-seq: RNA sequencing
WGCNA: Weighted gene co-expression network analysis
HMDD: Human miRNA Disease Database
ROC: Receiver operating characteristic
qRT-PCR: Quantitative real-time polymerase chain reaction
SNc: Substantia nigra compact
DaTSCAN: DaT-SPECT imaging with (^123^I) ioflupane
CSF: Cerebrospinal fluid
NFL: Neurofilament light
NDDs: Neurodegenerative diseases
CNS: Central nervous system
AUC: Area under the curve
TEM: Transmission electron microscopy
PIGD: Postural instability gait disorder
