# Supplementary material for: Several miRNAs derived from serum extracellular vesicles are potential biomarkers for early diagnosis and progression of Parkinson’s disease
Source: Transl Neurodegener. 2021 Jul 28;10:25. doi: 10.1186/s40035-021-00249-y (PMC8317324; doi:10.1186/s40035-021-00249-y)
Supplement: Supplementary file 1 — Additional file 1: Fig. S1. The degree of miRNA functional enrichment in stages II–III. Fig. S2. ROC curve results of 10 miRNAs that did not show significant diagnostic value. Fig. S3. qRT-PCR results of 10 miRNAs whose specific expression failed to be verified by this analysis. Fig. S4. Venn diagram of miRNAs validated by ROC curve and qRT-PCR. Fig. S5. The expression of miRNAs validated by ROC curves and qRT-PCR assay, in the sera of PD participants. Table S1. Primer sequences for qRT-PCR. [file 40035_2021_249_MOESM1_ESM.pdf]

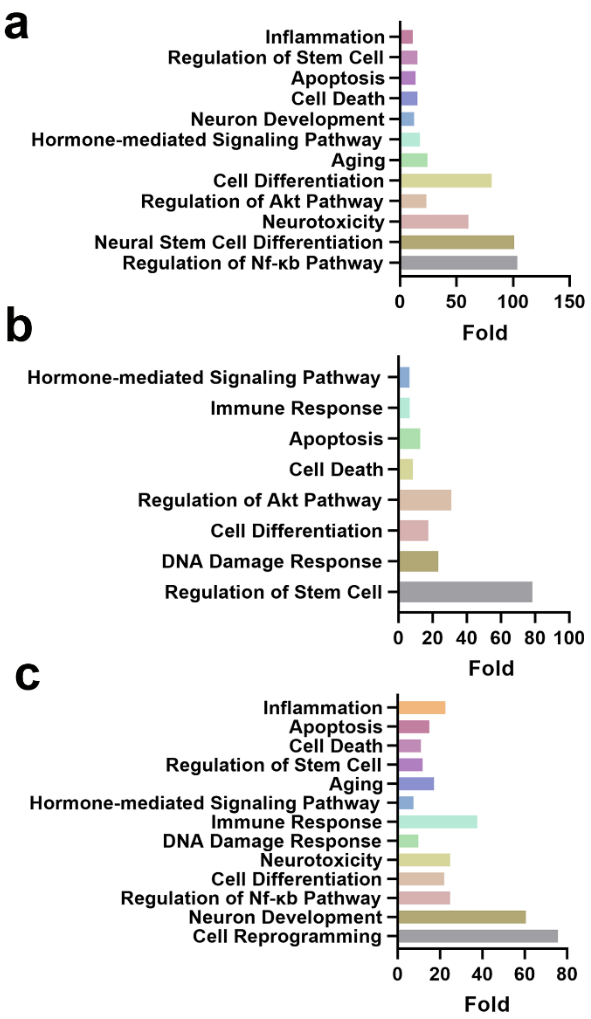

Fig. S1. The degree of miRNA functional enrichment in stages II–III.

(**a**). The degree of miRNA functional enrichments in stage II.

(**b**). The degree of miRNA functional enrichments in stage III.

(**c**). The degree of miRNA functional enrichments in stage IV.

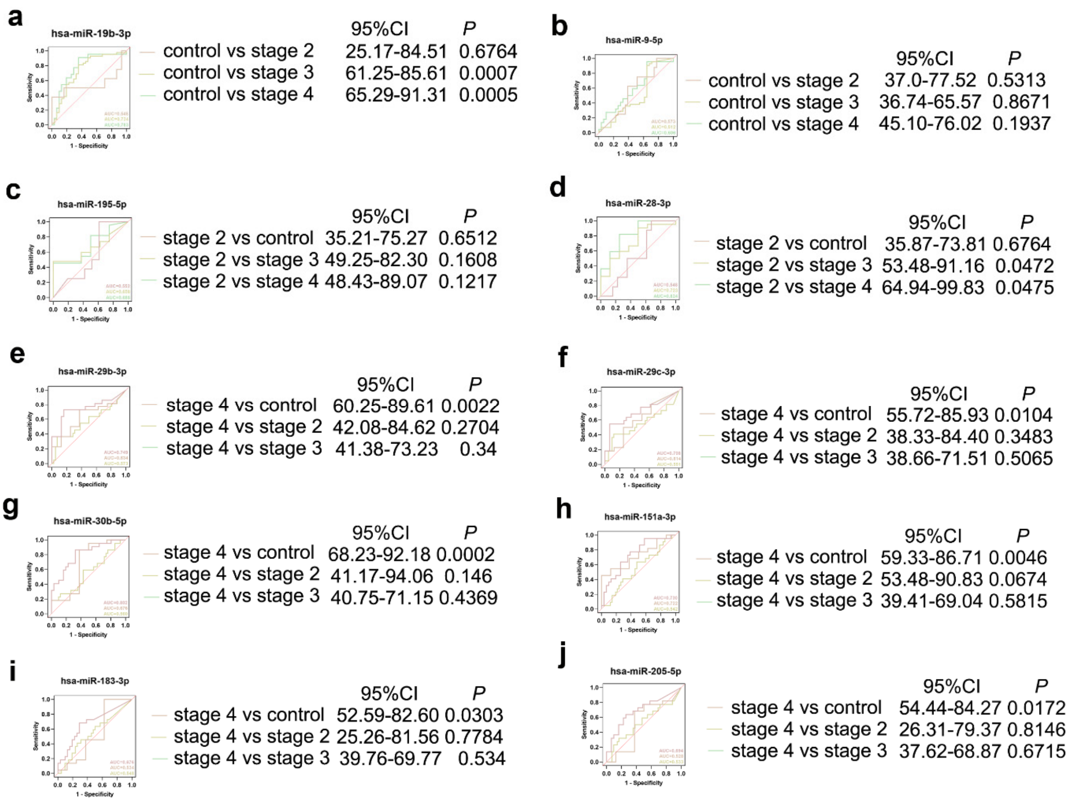

Fig. S2. ROC curve results of 10 miRNAs that did not show significant diagnostic value.

(**a**). ROC curves of hsa-miR-19b-3p differentiated stage II, III and IV from control.

(**b**). ROC curve of hsa-miR-9-5p differentiated stage II, III and IV from control.

(**c**). ROC curve of hsa-miR-195-5p differentiated stage II from control, stage III and stage IV.

(**d**). ROC curve of hsa-miR-28-3p differentiated stage II from control, stage III and stage IV.

(**e**). ROC curve of hsa-miR-29b-3p differentiated stage IV from control, stage II and stage III.

(**f**). ROC curve of hsa-miR-29c-3p differentiated stage IV from control, stage II and stage III.

(**g**). ROC curve of hsa-miR-30b-5p differentiated stage IV from control, stage II and stage III.

(**h**). ROC curve of hsa-miR-151a-3p differentiated stage IV from control, stage II and stage III.

(**i**). ROC curve of hsa-miR-183-3p differentiated stage IV from control, stage II and stage III.

(**j**). ROC curve of hsa-miR-205-5p differentiated stage IV from control, stage II and stage III.

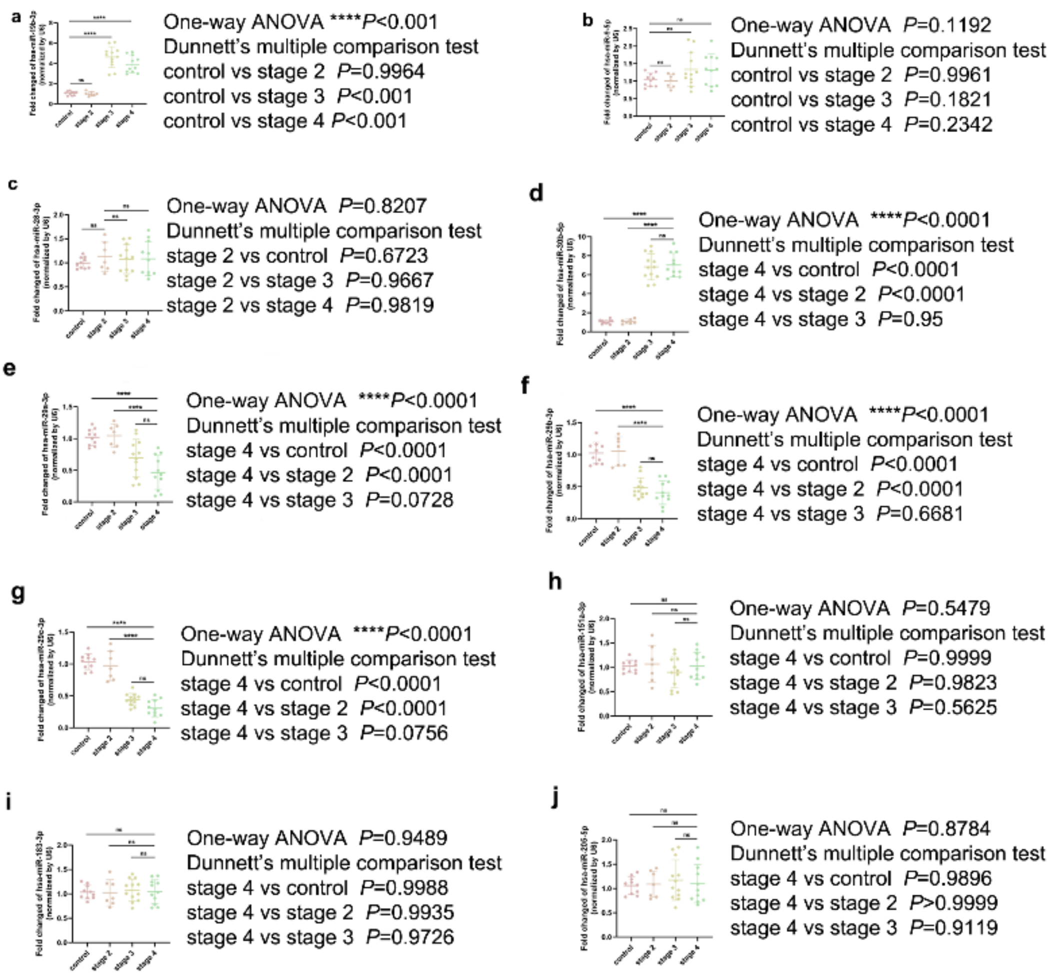

Fig. S3. qRT-PCR results of 10 miRNAs whose specific expression failed to be verified by this analysis.

(**a**). The expressions of hsa-miR-19b-3p in stage II, stage III and stage IV.

(**b**). The expressions of hsa-miR-9-5p in stage II, stage III and stage IV.

(**c**). The expressions of hsa-miR-28-3p in stage II, stage III and stage IV.

(**d**). The expressions of hsa-miR-30b-5p in stage II, stage III and stage IV.

(**e**). The expressions of hsa-miR-29a-3p in stage II, stage III and stage IV.

(**f**). The expressions of hsa-miR-29b-3p in stage II, stage III and stage IV.

(**g**). The expressions of hsa-miR-29c-3p in stage II, stage III and stage IV.

(**h**). The expressions of hsa-miR-151a-3p in stage II, stage III and stage IV.

(**i**). The expressions of hsa-miR-183-3p in stage II, stage III and stage IV.

(**j**). The expressions of hsa-miR-205-5p in stage II, stage III and stage IV. Data were analyzed by one-way ANOVA with Dunnett's multiple comparisons test (mean ± SD; $n = 40$, ****$P < 0.0001$).

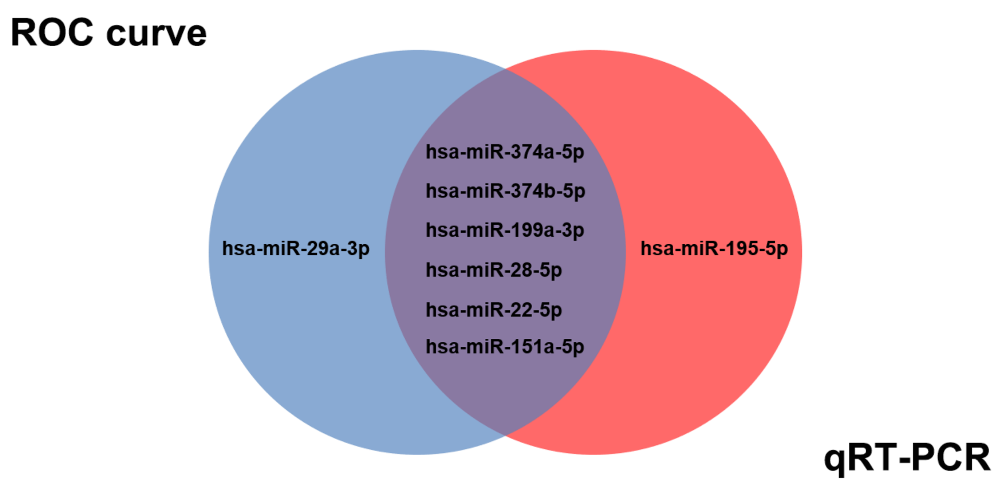

Fig. S4. Venn diagram of miRNAs validated by ROC curve and qRT-PCR.

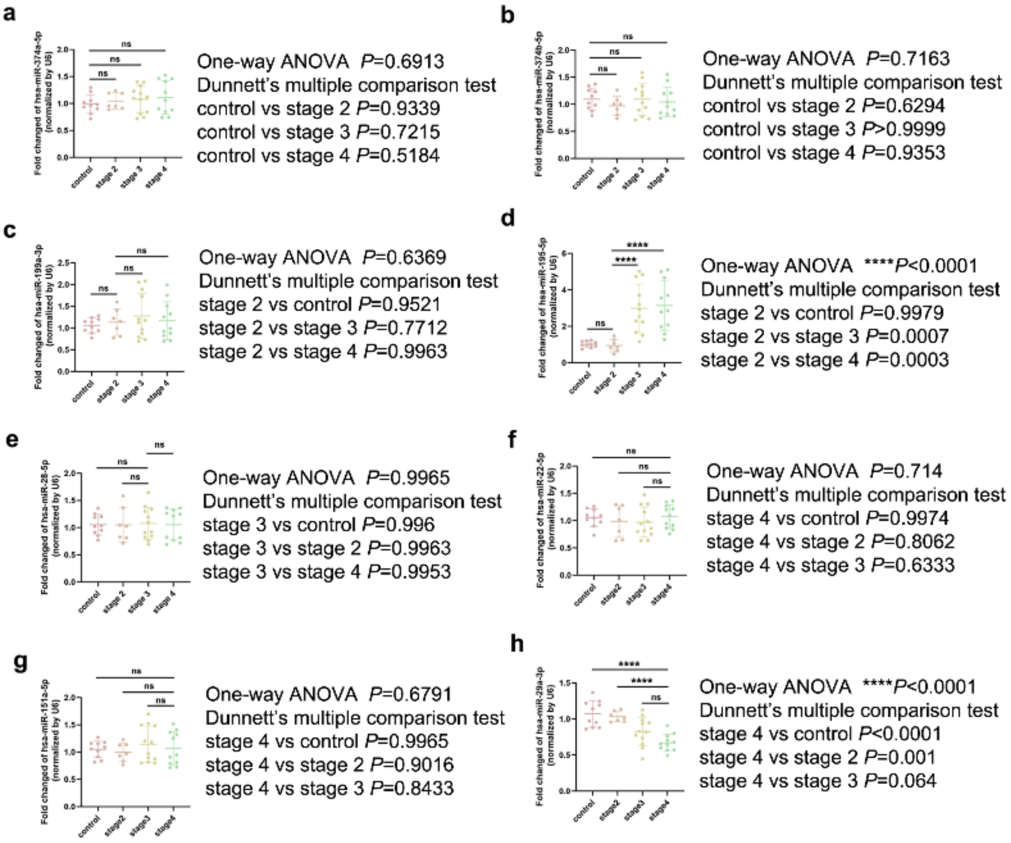

Fig. S5. The expression of miRNAs validated by ROC curves and qRT-PCR assay, in the sera of PD participants.

(**a**). The expressions of hsa-miR-374a-5p in stage II, stage III and stage IV.

(**b**). The expressions of hsa-miR-374b-5p in stage II, stage III and stage IV.

(**c**). The expressions of hsa-miR-199a-3p in stage II, stage III and stage IV.

(**d**). The expressions of hsa-miR-195-5p in stage II, stage III and stage IV.

(**e**). The expressions of hsa-miR-28-5p in stage II, stage III and stage IV.

(**f**). The expressions of hsa-miR-22-5p in stage II, stage III and stage IV.

(**g**). The expressions of hsa-miR-151a-5p in stage II, stage III and stage IV.

(**h**). The expressions of hsa-miR-29a-3p in stage II, stage III and stage IV. Data were analyzed by one-way ANOVA with Dunnett's multiple comparisons test (mean ± SD; $n = 40$; **** P <0.0001).

Table S1. Primer sequences for qRT-PCR.

Table S1 The primer sequences used by qRT-PCR

miRNA	Primer sequences	
U6	5'-GCTCGCTTCGGCAGCA-3'	(forward)
U6	5'-AACGCTTCACGAATTTGCGT-3'	(reverse)
hsa-miR-374a-5p	5'-TTATAATACAACCTGATAAGTG-3'	(forward)
hsa-miR-374b-5p	5'-ATATAATACAACCTGCTAAGTG-3'	(forward)
hsa-miR-19-3p	5'-TGTGCAAATCCATGCAAAACTGA-3'	(forward)
hsa-miR-30c-5p	5'-TGTAAACATCCTACACTCTCAGC-3'	(forward)
hsa-miR-199a-3p	5'-ACAGTAGTCTGCACATTGGTTA-3'	(forward)
hsa-miR-195-5p	5'-TAGCAGCACAGAAATATTGGC-3'	(forward)
hsa-miR-5100	5'-TTCAGATCCCAGCGGTGCCTCT-3'	(forward)
hsa-miR-28-5p	5'-AAGGAGCTCACAGTCTATTGAG-3'	(forward)
hsa-miR-378a-5p	5'-CTCCTGACTCCAGGTCCTGTGT-3'	(forward)
hsa-miR-22-5p	5'-AGTTCTTCAGTGGCAAGCTTTA-3'	(forward)
hsa-miR-151a-5p	5'-TCGAGGAGCTCACAGTCTAGT-3'	(forward)
hsa-miR-4443	5'-TTGGAGGCGTGGGTTTT-3'	(forward)
hsa-miR-30b-5p	5'-TGTAAACATCCTACACTCAGCT-3'	(forward)
hsa-miR-29a-3p	5'-TAGCACCATCTGAAATCGGTTA-3'	(forward)
hsa-miR-29b-3p	5'-TAGCACCATTTGAAATCAGTGTT-3'	(forward)
hsa-miR-29c-3p	5'-TAGCACCATTTGAAATCGGTTA-3'	(forward)
hsa-miR-205-5p	5'-TCCTTCATTCCACCGGAGTCTG-3'	(forward)
hsa-miR-183-3p	5'-GTGAATTACCGAAGGGCCATAA-3'	(forward)
hsa-miR-21-5p	5'-TAGCTTATCAGACTGATGTTGA-3'	(forward)
hsa-miR-25-5p	5'-AGGCGGAGACTTGGGCAATTG-3'	(forward)
hsa-miR-1260b	5'-ATCCCACCACTGCCACCAT-3'	(forward)
hsa-miR-151a-3p	5'-CTAGACTGAAGCTCCTTGAGG-3'	(forward)
hsa-miR-9-5p	5'-TCTTTGGTTATCTAGCTGTATGA-3'	(forward)
hsa-miR-28-3p	5'-CACTAGATTGTGAGCTCCTGGA-3'	(forward)
hsa-miR-4492	5'-GAGGCTGAGCGCTCGTCA-3'	(forward)

The downstream primers of miRNAs come from universal primers in the kit (miScript II RT kit, QIAGEN) except U6.
